# Effects of PLA Film Incorporated with ZnO Nanoparticle on the Quality Attributes of Fresh-Cut Apple

**DOI:** 10.3390/nano7080207

**Published:** 2017-07-31

**Authors:** Wenhui Li, Lin Li, Yun Cao, Tianqing Lan, Haiyan Chen, Yuyue Qin

**Affiliations:** 1 Institute of Yunnan Food Safety, Kunming University of Science and Technology, Kunming 650550, China; 15559823733@163.com (W.L.); caoyundyx@163.com (Y.C.); lantianqing_8019@163.com (T.L.); seacome@163.com (H.C.); 2 College of Light Industry and Food Science, South China University of Technology, Guangzhou 510640, China; felinli@scut.edu.cn

**Keywords:** fresh-cut apple, PLA, nano-ZnO, nano-blend, microbial analyses

## Abstract

A novel nanopackaging film was synthesized by incorporating ZnO nanoparticles into a poly-lactic acid (PLA) matrix, and its effect on the quality of fresh-cut apple during the period of preservation was investigated at 4 ± 1 °C for 14 days. Six wt % cinnamaldehyde was added into the nano-blend film. Scanning electron microscope (SEM) analysis showed a rougher cross-section of the nano-blend films and an X-ray diffraction (XRD) was carried out to determine the structure of the ZnO nanoparticles. Compared to the pure PLA film, the nano-blend film had a higher water vapor permeability (WVP) and lower oxygen permeability. With the increase of the nanoparticles (NPs) in the PLA, the elongation at break (ε) and elastic modulus (EM) increased, while tensile strength (TS) decreased. Thermogravimetric analysis (TGA) presented a relatively good thermostability. Most importantly, the physical and biochemical properties of the fresh-cut apple were also measured, such as weight loss, firmness, polyphenol oxidase (PPO), total phenolic content, browning index (BI), sensory quality, and microbiological level. The results indicated that nano-blend packaging films had the highest weight loss at the end of storage compared to the pure PLA film; however, nanopackaging provided a better retention of firmness, total phenolic countent, color, and sensory quality. It also had a remarkable inhibition on the growth of microorganisms. Therefore, Nano-ZnO active packaging could be used to improve the shelf-life of fresh-cut produce.

## 1. Introduction

The consumption of fresh-cut vegetables and fruits has become more and more popular over the past decades. The phenomenon is caused by the greater consumer interest in healthy and nutritious diets and the changes in their lifestyles [1]; for instance, fresh-cut apples have recently become popular snacks in food service establishments, for family consumption, and for school lunch programs due to their antioxidants and other nutrient components [2]. However, the preservation of fresh-cut apples is difficult work, because the fresh-cut fruit undergoes rapid deteriorative processes which can promote the decay of the fruit. Meanwhile, because of enzymatic browning, tissue softening, and the microbial growth of the sliced fruits, they generally have a short shelf-life [3]. In order to extend the shelf-life of the fresh-cut apple, a range of treatments have been applied, such as the use of natural browning inhibitors [4], salt and chemical treatments [5], coating agents and reduced oxygen atmospheres [6,7].

In recent years, research on the production of innovative food packaging materials has received considerable attention because of the growing field of the preparation of advanced functional composites and nanocomposites [8]. Biopolymer-based films are usually used for the preparation of antimicrobial packaging systems, which contain the advantages of biopolymers and the antimicrobial properties of additives [9,10]. Poly-lactic acid (PLA) is one of the most extensively studied bio-based polyesters, which was derived from lactic acid monomer [11]. It is one of the polymers with the highest potential because of its superior mechanical properties, versatility, and low cost [12,13]. PLA has been approved by the United States Food and Drug Administration (FDA) for use in food-contact materials. However, because pure PLA has the flaws of inelasticity and brittleness, slow degradation, high crystallinity and costliness, the application of PLA is limited [14]. A large number of research methods, including the addition of modifiers, compatibilization, blending, and physical treatments, have been performed to improve its properties. Generally, incorporating PLA with other bioactive ingredients is known to be the most effective method to obtain a polymeric material with required properties. Cinnamaldehyde is derived from cinnamon and is a naturally occurring aromatic α,β-unsaturated aldehyde and has been certified by the Food and Agriculture Organization/World Health Organization (FAO/WHO) Expert Committee on Food Additives (JECFA) for use as a food-flavoring agent. Meanwhile, it is the major ingredient of cinnamon bark extract [15]. It has been approved that cinnamaldehyde can work against a broad spectrum of food-borne pathogens effectively, and it is a well-known natural antimicrobial compound [16].

Recently, new types of nano-inorganic antimicrobial materials have become widely used in many fields, because they are stable under high temperatures and pressure conditions, and because they are also generally considered to be safe for human beings and animals in comparison to organic substances. ZnO has been found to be used widely in daily life in applications such as medical devices, drug delivery, and cosmetics [17,18]. Lepo et al. found that polypropylene films which contains nano-ZnO had good mechanical and oxygen barrier properties [19]. Li et al. reported that low-density polyethylene nanocomposite packaging materials containing silver and ZnO nanoparticles were conducive in prolonging the shelf-life of fresh orange juice during storage at 4 °C [20]. Emamifar et al. successfully developed a novel polyvinyl chloride film containing nano-ZnO particles as active food packaging to improve the shelf-life of fresh-cut ‘Fuji’ apple [21]. However, a shortcoming of the use of nanoparticles in food packaging is that nanoparticles will migrate from the packaging materials toward the packaged food, which would impair human health and environmental safety. Our previous study had certificated that the migration amounts of the NPs (TiO_2_ and Ag) from the nano-blend film to cheese samples and food simulants were still far below the migration limit of 1 mg/kg as defined by EFSA for food contact materials [22,23]. These results indicated that PLA films with nanoparticles could be considered as a safe packaging material.

In this paper, the material characterization of the PLA/nano-ZnO blend film was first determined. Then, the effects of nano-blend packaging film on weight loss, tissue firmness, polyphenoloxidase (PPO), total phenol content, color, microbiological quality, and sensory attributes of the apples stored at 4 °C was evaluated. The aim was to provide appropriate potential technologies to decrease the undesirable physiological changes in fresh-cut apple and to extend its shelf-life.

## 2. Results and Discussion

### 2.1. Scanning Electron Microscope (SEM)

**Figure 1:**
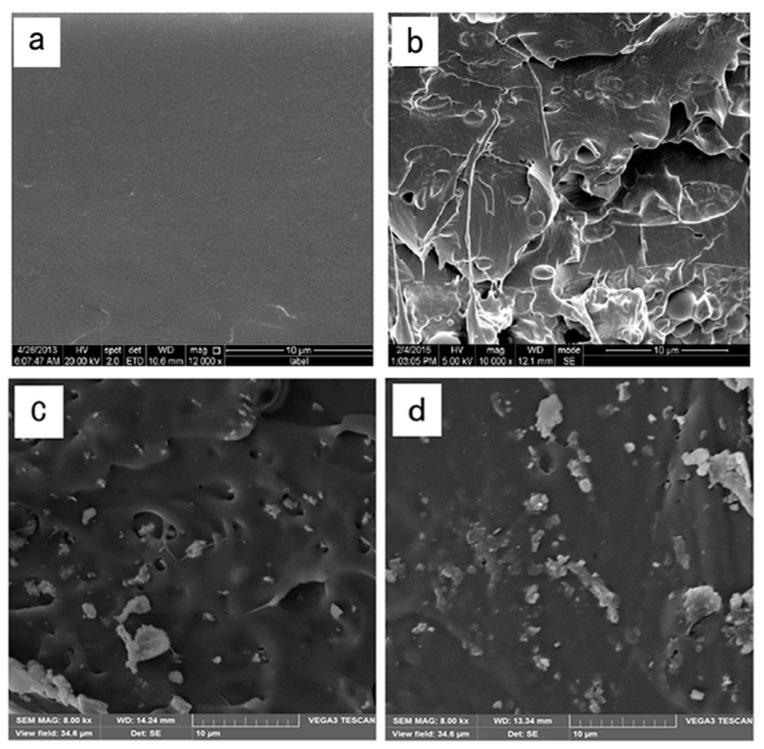
SEM micrographs of the fracture morphology of: (**a**) poly-lactic acid (PLA); (**b**) PLA/C6; (**c**) PLA/C6/ZnO1% and (**d**) PLA/C6/ZnO3%.

The cross-section morphology of all the films is shown in Figure 1. Figure 1a showed a smooth and flat appearance of the pure PLA film. This morphology can be explained by the brittle property of the PLA. The cross-section of the PLA/cinnamaldehyde blend films (Figure 1b) showed rougher surfaces and was heterogeneous with certain cavities and pores, which lead to a higher WVP of the film compared to the pure PLA. It can be seen from Figure 1c,d that the cross-section of PLA/nano-ZnO films showed rougher surfaces and that many voids were formed in the films. In the PLA/nano-ZnO blends with 1 wt % nano-ZnO, the nanoparticles were quite well distrubuted through the polymer matrix. When the loading of nano-ZnO increased up to 3 wt %, the distribution of nano-ZnO remained reasonably good, with only few nanofillers associating as small clusters. This might be because mineral surfaces covered with hydroxyl groups, such as ZnO ,are generally very receptive to bonding with alkoxysilanes [24].

### 2.2. X-ray Diffraction (XRD)

**Figure 2:**
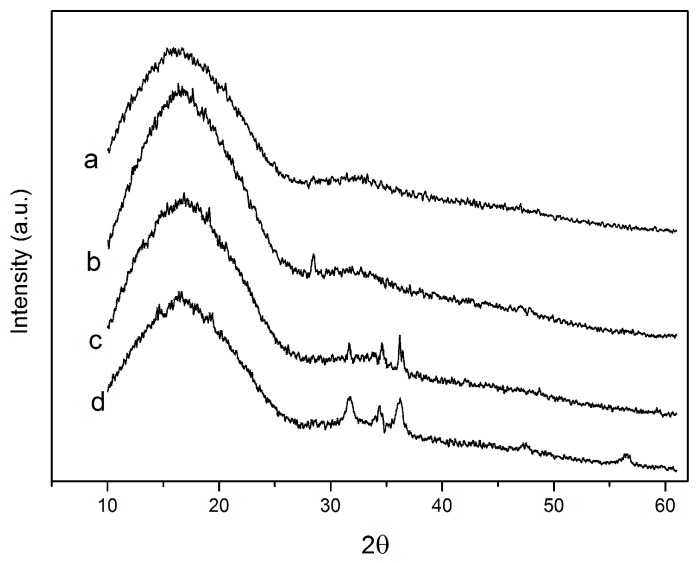
The XRD patterns of: (**a**) PLA; (**b**) PLA/C6; (**c**) PLA/C6/ZnO1% and (**d**) PLA/C6/ZnO3%.

Figure 2 shows the XRD patterns of neat PLA and all studied nanocomposite films. It can be seen from Figure 2 that all the films show a broad intensity, with a maximum appearing at approximately 2θ = 17°, which suggests that it is mainly an amorphous structure [25]. In addition, it can be easily seen from Figure 2c,d that the specific peaks were evidenced at 2θ = 31.6°, 2θ = 34.2° and 36.2°, which could be due to the diffraction planes of the crystalline form of ZnO, while their intensity clearly increased with nanofiller loading [26]. A similar result has been reported by Chu et al. and Pantani et al. [27,28].

### 2.3. Water Vapor Permeability (WVP)

The relative humidity in the packaging materials could have a great effect on the shelf-life of packaged products by their influence on microbial growth. Thus, the water vapor permeability (WVP) of the films were determined. The WVP of the four films are shown in Table 1. We can see from the Table 1 that the WVP of the two nano-blend films were significantly (*p* < 0.05) higher than the PLA/C6 and PLA film. This might be due to the hydrophilicity of nano-ZnO and the improved hydrophilic interaction of the films. In addition, we can observe from the SEM of the films that the morphological structures of PLA blend films changed, and that many voids in PLA/C6/ZnO blend films were formed, which allowed more water vapor transfer. However, with the increase of the nano-ZnO in the PLA film, the WVP decreased a small amount. This could be explained by the low particle diameter of the NPs, which would lead to a more tortuous pathway, reducing the diffusion coefficient [29]. A similar result has been obtained by Marra et al. [30].

### 2.4. Oxygen Permeability

It can be seen from Table 1 that the oxygen permeability of the nano-blend films was significantly (*p* < 0.05) lower than the PLA/C6 and PLA film, and that the oxygen permeability decreased with the increase of nano-ZnO content. The tortuous pathway prolongs the oxygen pathway and is the main reason for the improvement of oxygen resistance in the nano-blend films [31]. In addition, the oxygen permeability of the PLA/C6 film was higher than the pure PLA. This might be due to the higher amorphous phase of the PLA/C6 film compared to the pure PLA film; the higher amorphous phase makes the polymer more permeable [32]. This indicated that the PLA/C6 film had more amorphous phases than pure PLA film.

### 2.5. Mechanical Properties

The mechanical properties such as tensile strength (TS), elongation at break (ε) and elastic modulus (EM) of the films are listed in Table 2. Table 2 shows that the EM, TS and E of the pure PLA were 3027.79, 47.78% and 5.35%, respectively. With the addition of cinnamaldehyde, the PLA/C6 film had a higher E value and lower EM and TS, compared to the pure PLA. This result might be because the addition of cinnamaldehyde could lower the interaction between PLA molecules and hinder polymer chain-to-chain interactions. This would therefore lead to a significant decrease in the tensile strength and elasticity modulus of films [33]. Moreover, the discontinuities induced in the PLA matrix by cinnamaldehyde droplets would give the films a greater ability for deformation without breaking, as well as enhancing the E value [34]. In addition, compared with the PLA/C6 film, the EM and E of the two nanoblends were shifted to higher values and the TS was shifted to a lower value. This result might be due to the additions of nano-ZnO, which decreased the interactions between PLA chains and enhanced the mobility of PLA chains [35]. A similar result has been reported by Murariu et al. [36]. Lizundia et al. also reported that, with the increase of the ZnO nanoparticles in the PLLA, the elasticity modulus of the nanocomposite film was increased [37].

### 2.6. Opacity

**Figure 3:**
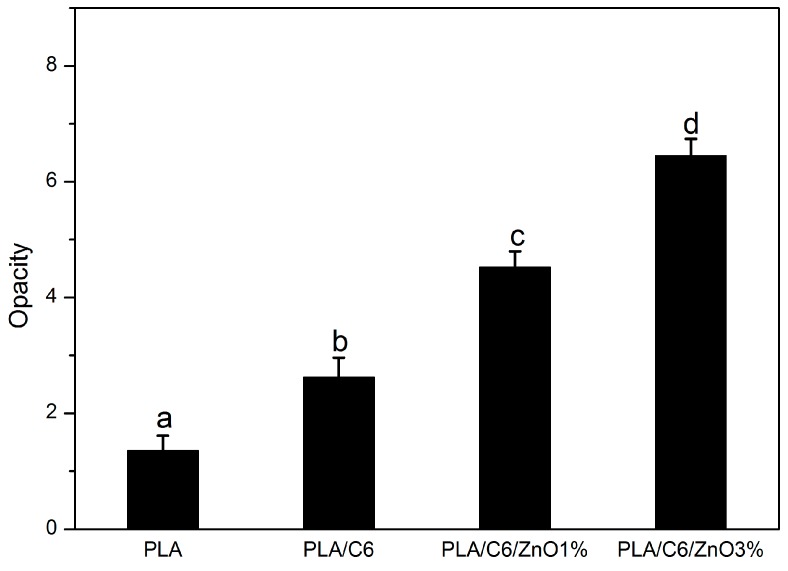
Opacity analysis of pure PLA and PLA nano-composite films. Values followed by different superscript letters (a–d) in the same column were significantly different (*p* < 0.05), where a is the lowest value.

**Figure 4:**
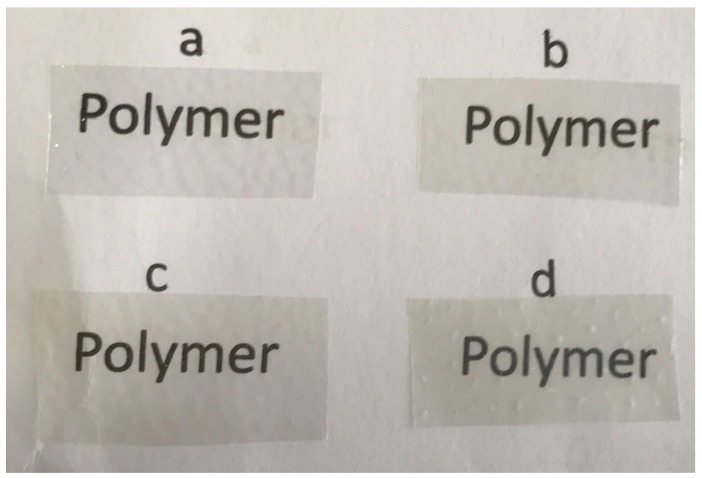
The visual appearance of: (**a**) PLA; (**b**) PLA/C6; (**c**) PLA/C6/ZnO1% and (**d**) PLA/C6/ZnO3%.

The opacity of all the films is shown in Figure 3, and the visual appearance of the films is shown in Figure 4. Figure 3 shows that the transparency of the PLA/C6 film decreased with the addition of the cinnamaldehyde. The distribution of essential oils in the polymer matrix may be relied on the distribution of essential oils in the polymer matrix. In addition, it can be seen in Figure 3 that the opacity of the film increased significantly (*p* < 0.05) as the addition of ZnO nanoparticles increased. This result was probably due to the color of the nano-ZnO powder. A similar result has been reported by Espitia et al. [38]. However, from Figure 4, it can be easily seen that the differences in transparency among the four film samples were not perceptible to the human eye; this result suggested that, for consumers, the nano-blend films still have a good transparency and that people would be able to see items sufficiently through the films.

### 2.7. Differential Scanning Calorimetry (DSC)

**Figure 5:**
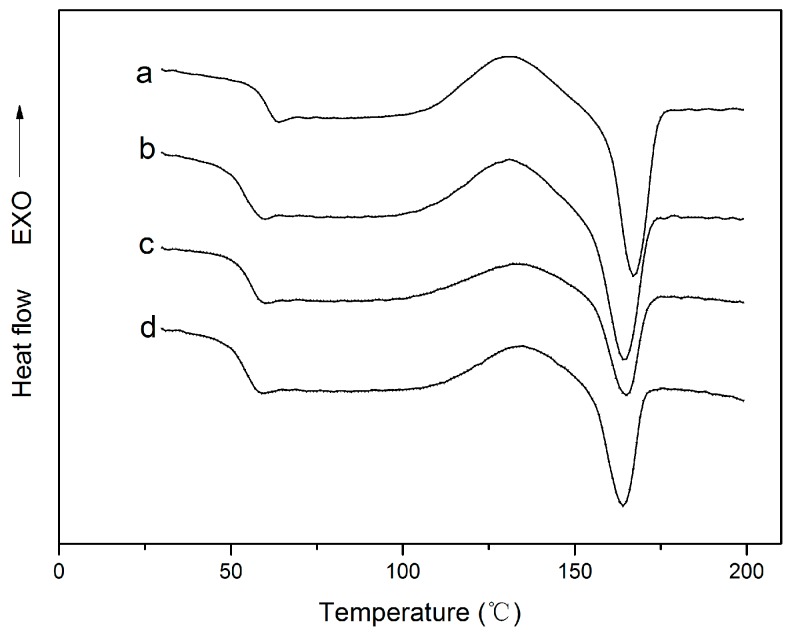
Differential scanning calorimetry (DSC) curves of: (**a**) PLA; (**b**) PLA/C6; (**c**) PLA/C6/ZnO1% and (**d**) PLA/C6/ZnO3%.

The typical DSC curves of all the four films are shown in Figure 5. The glass transition (*T_g_*), cold crystallization (*T_c_*), melting process (*T_m_*) and the crystallinity (*X_c_*) can be found in Table 3. It can be easily seen that, with the addition of cinnamaldehyde, the *T_g_* and the *T_m_* values of the PLA were decreased to lower levesl and the *T_c_* and *X_c_* value increased to higher levels. This might be due to the molecular structure of cinnamaldehyde, which changed the overall chain mobility of polymer matrix, resulting in faster crystallization kinetics in the blends [39]. However, Figure 5 showed that the *T_g_* and *T_m_* did not show significant modifications with the introduction of nano-ZnO particles. This phenomenon of enthalpic relaxation is typical for a polymeric material in the glassy state that undergoes physical ageing [40]. Murariu et al. prepared nanocomposites containing ZnO nanoparticles and found that the addition of those nanoparticles slightly decreases the glass transition temperature of a polymer matrix [41]. Lizundia also revealed that both glass transition temperature (*T_g_*) and melting temperature (*T_m_*) remain unchanged for all the PLA/ZnO nano-blend films [37]. Moreover, as listed in Table 3, an obvious increase of the degree of crystallinity (*X_c_*) of the PLA/nano-ZnO blends was found with the addition of the ZnO nanoparticles. This result can be explained by the phenomenon of heterogeneous nucleation.

### 2.8. Thermogravimetric Analysis (TGA)

**Figure 6:**
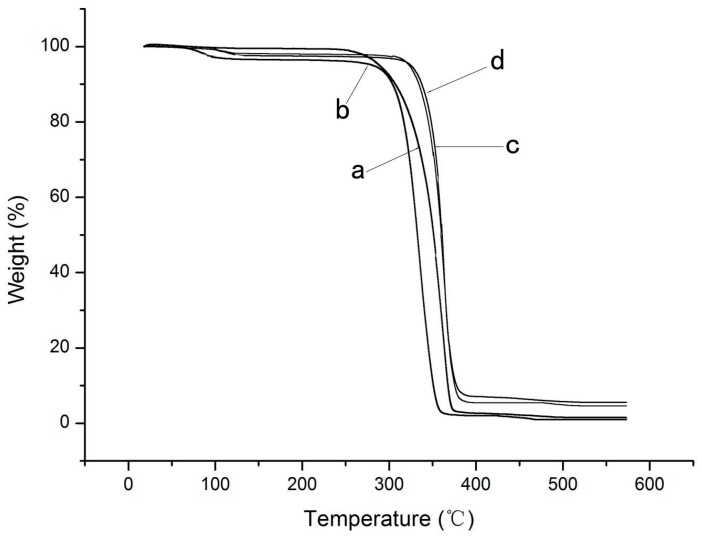
Thermogravimetric analysis (TGA) curves of the PLA/NPs blend films: (**a**) PLA; (**b**) PLA/C6; (**c**) PLA/C6/ZnO1%; (**d**) PLA/C6/ZnO3%.

The TGA curves of all the four films are shown in Figure 6. As shown in Figure 6, The PLA film decomposed in a single-step process with the onset of decomposition temperature (*T*_onset_) of 286 °C and the maximum decomposition temperature (*T*_dmax_) centered at 375 °C. This result indicated that there was no remaining dichloromethane in the film samples. The incorporation of the cinnamaldehyde in the PLA (Figure 6b) led to a two-step degradation process, where the first degradation step was 65 and 112 °C and the second degradation step was similar to the pure PLA film. The nano-blend film also had a two-step degradation process, and both steps of the two-step degradation was higher than for the PLA film. This could be explained by the evaporation of the cinnamaldehyde incorporated in the blends [42]. As can be seen from Figure 6, both *T*_onset_ and *T*_dmax_ of the the nano-blend film shifted to a higher value compared with the pure PLA film. This indicated that the addition of ZnO nanoparticles could obviously improve the thermal stability of PLA film. This might be due to the fact that TiO_2_ NPs acted as a heat barrier in the early stages of thermal decomposition [43].

### 2.9. Weight Loss

**Figure 7:**
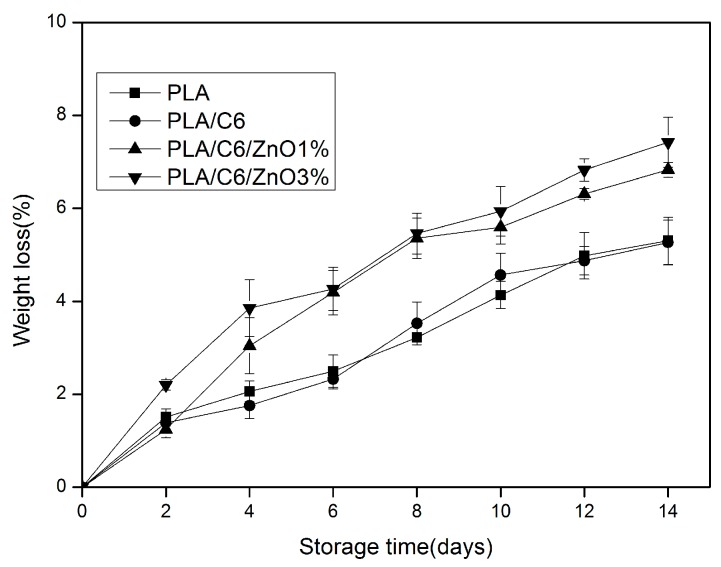
Effect of different packages on the weight loss of apple sample stored at 4 ± 1 °C for 14 days. Data are presented as mean ± standard deviation.

The weight loss values of the apple samples during storage are shown in Figure 7. During storage, all of the packaged samples increased in terms of weight loss; this was probably related to the continuous moisture movement from the apple slices to the surrounding environment. As shown in Figure 7, the weight loss of apple samples packed by PLA/C6/ZnO1% and PLA/C6/ZnO3% film was significantly (*p* < 0.05) higher than the PLA/C6 and PLA film after 4 days storage. A certain amount of nanoparticle embedded in the PLA could effectively improve the water vapour permeability of the PLA film. It can be seen from Table 1 that the apple samples were packed by film with different water vapor barrier abilities. At the end of storage, the maximum weight loss was recorded for samples packed in the PLA/C6/ZnO3% film; this reached 7.42% and was consistent with the WVP of the films. As shown in Figure 7, the weight loss of the sample packed by PLA/C6/ZnO3% film was a little higher than the sample packed by PLA/C6/ZnO1% over the whole storage time.

### 2.10. Tissue Firmness

**Figure 8:**
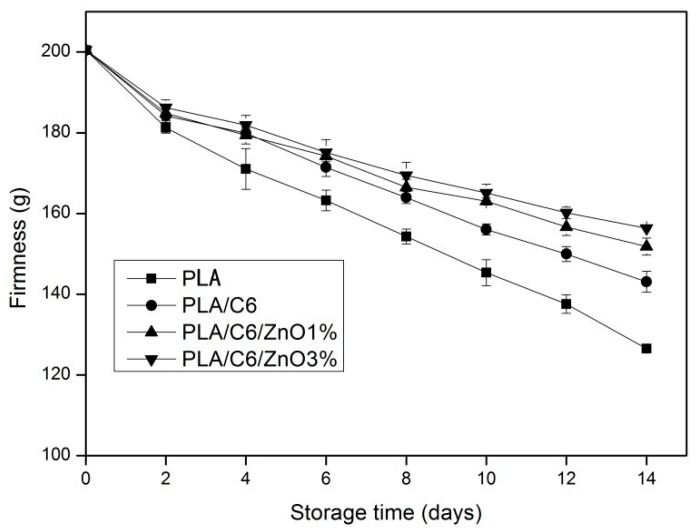
Effect of different packages on the firmness of apple sample stored at 4 ± 1 °C for 14 days. Data are presented as mean ± standard deviation.

Tissue firmness is one of the important indexes of the quality of fresh-cut apples. The trend of firmness-change of the apple samples packed by different packaging films is shown in Figure 8. All the samples showed a decreasing trend in firmness over the storage time. The highest level (156.33 g) was obtained with the PLA/C6/ZnO3% film and the lowest (126.53 g) was obtained with the PLA film. In addition, we can see from Figure 8 that the firmness-changes of samples packed by PLA/C6/ZnO1% and PLA/C6/ZnO3% film were significantly (*p* < 0.05) lower than the samples packed by the other two films after 6 days storage. This might be because the water vapor permeability of nano-blend films was higher than other films, and because packaging films with a low permeable rate can promote the relative humidity within the package, leading to a high metabolic activity and the speeding up of the process of apple softening [44].

### 2.11. Polyphenol Oxidase (PPO) Activity

**Figure 9:**
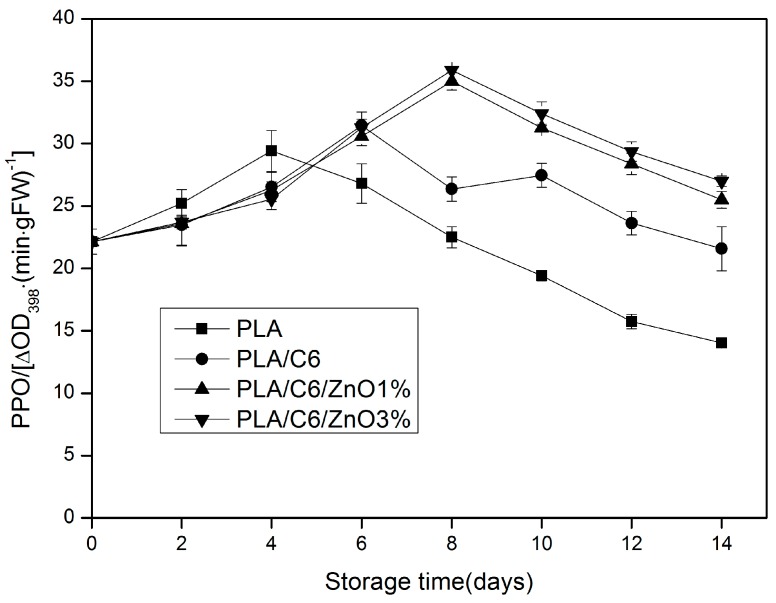
Effect of different packaging film on the polyphenoloxidase (PPO) activity of fresh-cut apples during storage at 4 ± 1 °C for 14 days. Data are presented as mean ± standard deviation.

PPO is usually considered as the most significant enzyme which causes the browning of post-harvest fruit and vegetable products [6]. It can be seen from Figure 9 that the activity of PPO gradually increased in the early stage and decreased in the late stage for all the samples. In addition, the pure PLA film had poorer oxygen barrier properties than PLA/nano-ZnO films, while the samples packed by the pure PLA film had a higher PPO activity during the first 4 days of storage due to the browning of the fresh-cut apple surface [45]. Then, the activity of PPO of the sample packed by PLA film decreases rapidly after the peak on the fourth day, because of the rapid tissue senescence of the apple. The PPO activity of the apple sample packed by the PLA/C6/nano-ZnO film was significantly (*p* < 0.05) higher than the apple sample packed by the PLA film and the PLA/C6 film after 6 days storage. This could be explained by the antibacterial property of the nanoparticle, which can delay the senescence of the apple. The high activity of PPO shows that the degree of tissue senescence was low; this can be used as a measure of the freshness of fresh-cut fruits and vegetables.

### 2.12. Total Phenolic Content

**Figure 10:**
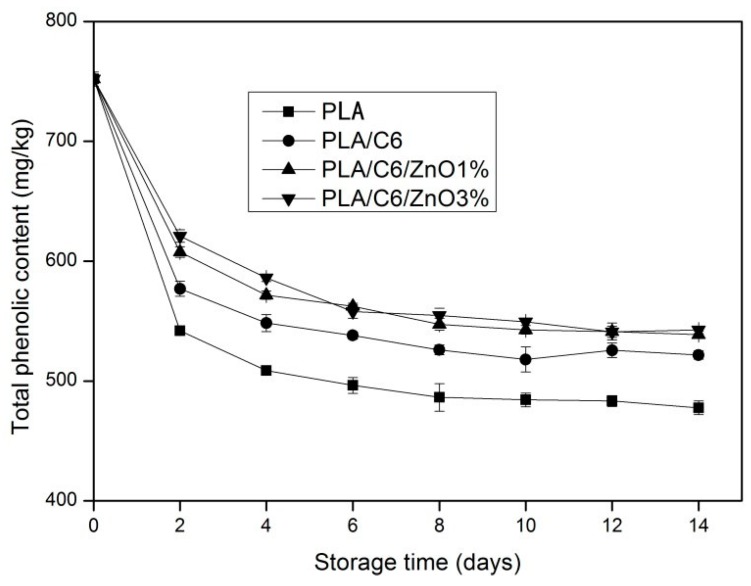
Effects of different packaging film on the total phenolic content of fresh-cut apples during storage at 4 ± 1 °C for 14 days. Data are presented as mean ± standard deviation.

The total phenolic content of the apple sample during storage is shown in Figure 10. The initial total phenol content of the samples before storage was 752 mg·kg^−1^. The total phenolic content of all the samples continually decreased during the first 6 days storage, and then tended to be smooth for the rest of the days. At the end of the storage, the total phenolic of the apple sample was at a range of 477–542 mg·kg^−1^. Similarly, Chen, C., et al. and Cocci et al. all reported that there was a sharp decline in the total phenolic content in fresh-cut apples during the first 2 days of storage [45,46]. This might be due to the fast oxidation of total phenols on the cut surface. The balance between total phenols and brown compounds was rapidly reached due to the PPO action. It can be seen from Figure 10 that the total phenolic content of apple samples packed by the PLA/C6/ZnO1% film and the PLA/C6/ZnO3% film was significantly (*p* < 0.05) higher than the sample packed by the other two films after 4 days of storage. The antibacterial properties of the nanoparticles could reduce the growth of microorganisms effectively, and the aging of the samples can be delayed.

### 2.13. Color

**Figure 11:**
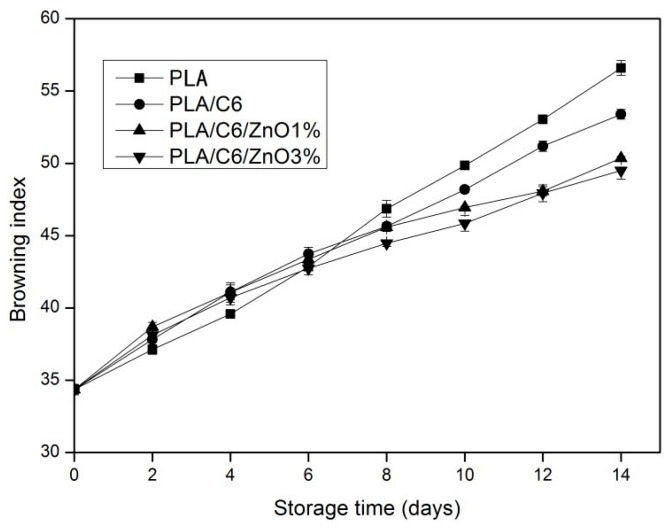
Effect of different packages on the browning index (BI) value of apple stored at 4 ± 1 °C for 14 days. Data are presented as mean ± standard deviation.

The browning index shows the purity of the brown colour; in addition, it is regarded as an important parameter during the process of enzymatic and nonenzymatic browning [21]. It can be seen from Figure 11 that the BI of apple slices increased gradually during storage, and that the BI value of the samples increased from 34.36 to a range of 49.51–56.60 after 14 days storage. The samples packed by PLA/C6/ZnO1% and PLA/C6/ZnO3% film were observed to have the lowest BI at the end of the storage compared to other samples, and there was no significant (*p* > 0.05) difference between them. Over the first 4 days of storage, the BI of the apple sample packed by the PLA was significantly (*p* < 0.05) lower than for the other three packagings, which could be due to the fact that the blend film has a higher water loss rate than the PLA film, and the surface desiccation of the sample packed by blend films. However, after 8 days of storage, the BI of the sample packed by the PLA and PLA/C6 film was significantly (*p* < 0.05) higher than the other two packagings; this may be on account of the rapid growth of the micro-organisms of the apple sample packed by the PLA and PLA/C6 films.

### 2.14. Microbial Analyses

**Figure 12:**
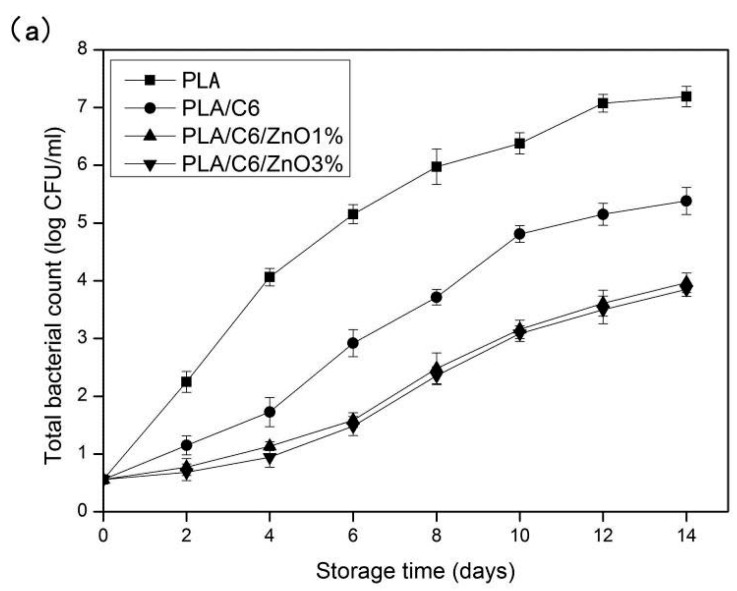

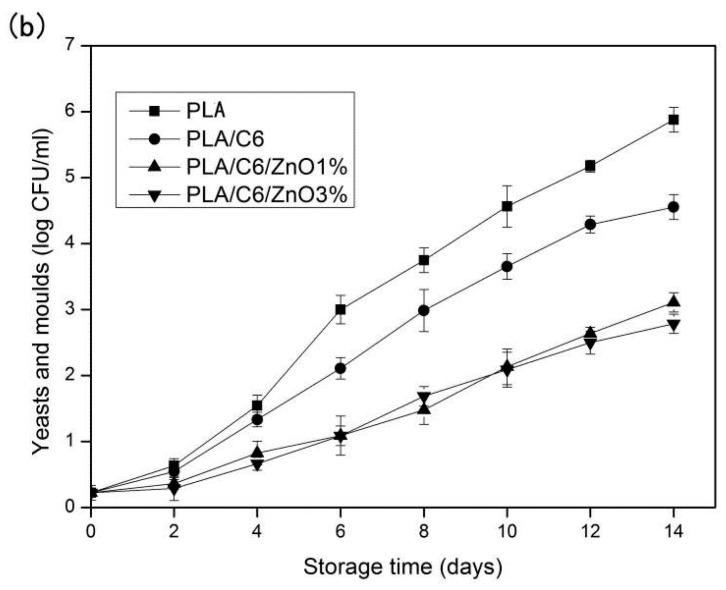
Effect of different packages on the total bacterial counts (**a**) and yeast–mold counts (**b**) of the fresh-cut apple stored at 4 ± 1 °C for 14 days. Data are presented as mean ± standard deviation.

Total bacterial, yeast and molds were considered as simple indicators of the general hygienic condition of the fresh-cut fruits. The changes in the count of total bacterial, yeast and molds of fresh-cut apples during 14 days of storage are shown in Figure 12. Microbial counts in the apple sample increased gradually during the 14 days of storage. As can be seen from Figure 12a, with the extension of storage days for fresh-cut apples treated with PLA film and PLA/C6 film, the total bacterial was significantly (*p* < 0.05) higher than that of the nano-blend film. The incorporation of the ZnO nanoparticles of PLA could effectively inhibit the growth of micro-organisms in fresh-cut apple. However, there was no significant (*p* > 0.05) difference in total bacterial count between PLA/C6/ZnO1% film and PLA/C6/ZnO3% film over the 14 days of storage. Because the nanofiller content and the state of dispersion are very important factors determining the antimicrobial ability of nano-blend packaging films, Sogvar, O.B., et al. reported that the minimum ZnO content required for effective microbial growth inhibition was 0.5 wt % [18]. Moreover, the total bacterial count of the apple sample packed by the PLA/C6 was also significantly (*p* < 0.05) lower than the sample packed by the PLA film, due to the antibacterial property of the cinnamaldehyde.

Yeasts and molds are important microbial contaminants in dairy products. It can be seen from Figure 12b that yeasts and molds in all groups increased over time. PLA/C6/ZnO1% and PLA/ZnO3% films were significantly (*p* < 0.05) more efficient than PLA and PLA/C6 film in inhibiting the growth of yeasts and molds. The yeast–mold count of the apple samples packed by PLA film and PLA/C6 film sharply increased and was higher than 5 log cfu/g after 14 days of storage. However, apple samples packed by PLA/C6/ZnO1% and PLA/C6/ZnO3% film only reached a population above 3.1 and 2.7 log cfu/g at the end of storage period, respectively. On the other hand, the yeast–mold count of the apple samples packed by PLA/C6 film was significantly (*p* < 0.05) lower than the sample packed by PLA film over the storage time, because there was no antimicrobial activity in the pure PLA film. Similar results on the inhibition of yeasts and moulds growth by blend packaging materials containing ZnO nanoparticles have been reported for fresh orange juice during cold storage (4 °C) by Emamifar, A., et al. [20]. Alkaladi et al. have certificated the antidiabetic activity of Zinc Oxide nanoparticles [47].

The antibacterial mechanism of zinc oxide has not yet been conclusive [48], There are three kinds of arguments regarding the antibacterial mechanism: (1) the liberation of antimicrobial ions of Zn^2+^ ions which directly contacts with cell walls results in destructing bacterial cell integrity [48,49]; (2) Reactive oxygen species (ROS) formation leading to the induction of oxidative stress which can, in particular, produce interior or out of cell H_2_O_2_ which leads to the death of micro-organisms [48,50]; (3) the accumulation of ZnO nanoparticles in the bacterial membrane causes the cellular internalization and membrane disorganization of the micro-organism.

### 2.15. Sensory Evaluation

Sensory evaluations such as odor, color, texture, and the overall acceptability of fresh-cut apple were examined on a nine-point hedonic scale. The sensory scores of fresh-cut apple are listed in Table 4. The values for all considered parameters decreased with increasing storage time for all groups. There was no significant (*p* > 0.05) difference in sensory scores for all packaging treatments on the second day; however, the odor, texture, and overall acceptability scores of the apple sample packed by PLA/C6, PLA/C6/ZnO1% and PLA/C6/ZnO3% film were significantly higher than that packed by PLA film after eight days of storage. At the end of the storage time, the overall acceptability scores of the sample packed by the PLA/C6/ZnO1% and PLA/C6/ZnO3% film were still higher than five, and the samples maintained proper characteristics. In term of the color, the samples packed by the PLA and PLA/C6 film were significantly (*p* < 0.05) higher than the other two films after the first eight days of storage; this might be because ZnO nanoparticles provoked a change in the apple surface color after a few days of storage, which is consistent with the changing trend of BI. Odor and texture were very important sensory parameters for fresh-cut apple, which affected the overall acceptability. It can be seen from Table 4 that the overall acceptability of fresh-cut apple was strongly affected by odor and texture, and that they have the same changing trend of scores.

Considering a score of five as corresponding to good and the limit of marketability, the fresh-cut apple sample packed by PLA, PLA/C6, PLA/C6/ZnO1% and PLA/C6/ZnO3% film achieved a shelf-life of 6, 10, 14 and 14 days, respectively. The results suggested that PLA/ZnO film could improve the quality of fresh-cut apple during refrigerated storage.

## 3. Materials and Methods

### 3.1. Technology Roadmap

**Figure 13:**
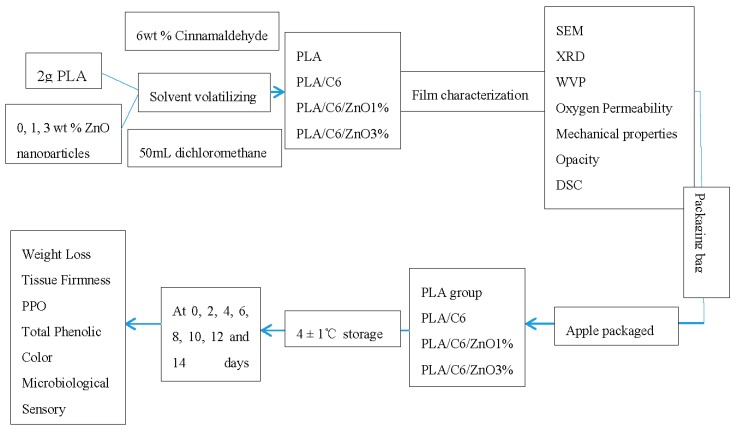
The technology roadmap for the whole study.

The technology roadmap of this study was shown in Figure 13. As the Figure 13 showed, preparing the nano-blend films was the first step, and the following was to determine the material characterization of the films. The nano-blend films were used to package the fresh-cut apples, so as to further studies on the preservation performance of the films. In the end, it investigated the physical and biochemical properties of the apple at different stages to evaluate the preservation performance of the films.

### 3.2. Materials

The PLA (M*w* = 280 kDa, M*w*/Mn = 1.98) used in this work was obtained from Natureworks LLC (Lincoln, NE, USA). Nano-ZnO powder with purity of 99.9% was obtained from MaiKun Industrial Co., Ltd. (Shanghai, China). Cinnamaldehyde was purchased from ZhanYun Co., Ltd. (Shanghai, China). Dichloromethane was obtained from Chengdu Kelong Chemical Co., Ltd. (Chengdu, China). Yunnan “ZhaoTong” apple was purchased in a local market (Kunming, China).

### 3.3. Film Preparation

Pure PLA, PLA/6 wt % cinnamaldehyde, PLA/6 wt % cinnamaldehyde/1 wt % nano-Zno and PLA/6 wt % cinnamaldehyde/3 wt % nano-Zno blend films were prepared by the solvent volatilizing method, which was similar to Qin et al. [51]. Brifely, 2 g PLA and 6 wt % cinnamaldehyde were dissolved in 50 mL dichloromethane. Then, 0 wt %, 1 wt % and 3 wt % nano-ZnO were added to the PLA/cinnamaldehyde dichloromethane solution respectively and stirred by magnetic stirrer for 10 h. The homogeneous PLA/cinnamaldehyde/nano-ZnO suspension was poured onto a polytetrafluoroethylene dish of 200 mm × 200 mm and dried in a vacuum oven at ambient temperature for overnight. The PLA film with 6 wt % cinnamaldehyde was named as PLA/C6 film. The PLA film with 6 wt % cinnamaldehyde and 1 wt % nano-ZnO was named as PLA/C6/ZnO1% nano-blend film. The PLA film with 6 wt % cinnamaldehyde and 3 wt % nano-ZnO was named as PLA/C6/ZnO3% nano-blend film. Pure PLA was used as control.

### 3.4. Scanning Electron Microscopy (SEM) of the Film

The cross-section morphology of the films was performed by scanning electron microscopy (S-3400N, Hitachi Ltd., Tokyo, Japan). Before the observation, the films were submerged in liquid nitrogen and broken, and then the films needed to be coated with a thin conductive gold layer in 20 nm thick. The method was similar to our previous work [51].

### 3.5. X-ray Diffraction (XRD)

The XRD analysis of the films were performed by using a an X-ray diffractometer (D8 Advance, Brucker, Karlsruhe, Germany) with Cu Kα radiation, at a voltage of 40 kV and an electricity of 40 mA. The samples were scanned in the diffraction angle 2θ, with a scan speed of 2°/min at room temperature.

### 3.6. Water Vapor Permeability (WVP) of the Film

Based on the ASTM E96-95 standard method, the WVP of the films was determined by gravimetry [52]. Briefly, the top of the measuring cups with desiccants were covered by the films. The covered bottles were put into constant temperature and humidity chambers with a temperature of 20 °C and relative humidity of 50%; then, the weight loss of each bottle was measured hourly for 12 h. The WVP of the film was calculated with the following formula [51]:

WVP = (WVTR × *L*)/∆*P* (1)

where WVTR is the water vapor transmission rate (g/m^2^ s) through the film, *L* is the average film thickness (m), and Δ*P* is the water vapor pressure difference (Pa) between the two sides of the film. This test was conducted in triplicated for film.

### 3.7. Oxygen Permeability

The oxygen permeability of the films was determined by a non-invasive oxygen analyzer system (OxySense, Inc., Dallas, TX, USA) equipped with high purity nitrogen and oxygen. The oxygen transmission rate (OTR) test of the film consists of determining the amount of oxygen that passes through the surface (7.66 cm^2^) of the film, in 24 h, at the temperature of 23 °C [30].

The permeability is obtained by multiplying the OTR [cm^3^/(m^2^ × 24 h)] for the film thickness (cm) and dividing by the difference of partial pressure (bar) present in the two chambers.

Oxygen Permeability = OTR × (thickness/ΔP) = [cm^3^/(m^2^ × 24 h)] × (cm/bar) (2)

### 3.8. Mechanical Properties

The mechanical properties of the films were tested by using CMT 4104 tensile testing equipment (MTS Systems Co., Ltd., Shanghai, China). The initial grip separation was set at 100 mm and the crosshead speed was set at 50 mm/min according to ASTM D638. An average of six test values were taken for each sample.

### 3.9. Opacity

The opacity of the films were evaluated by using a UV-vis spectrophotometer (T90, Beijing Purkinje General Instrument Co., Ltd., Beijing, China) to measure the absorbance at 600 nm [53]. Briefly, the film sample was cut into a rectangle section (0.7 cm × 1.5 cm), and then placed it in the spectrophotometer test cell. All measurements were performed in triplicate.

### 3.10. Differential Scanning Calorimetry (DSC)

The thermal behaviors of all the films were evaluated by a TA Instruments (DSC 214, Netzsch, Selb, Germany) under an inert nitrogen stream. About 10 mg of specimen was sealed in an aluminum pan and the DSC scans were heated from 10 to 200 °C at a heating rate of 20 °C/min, then cooled to 20 °C. The second heating scan was used to evaluate the glass transition temperature (*T*_g_), melting temperature (*T_m_*) and cold crystallization temperature (*T_c_*). In addition, the percentage of crystallinity (*X_c_*) was calculated according to the following equation:

(3) $$X_{c}\left(\%\right)=\frac{\Delta H_{m}-\Delta H_{c}}{\Delta H_{m}^{o}\times w}\times 100$$

where Δ*H_m_* is the melting enthalpy (J/g) of PLA in the sample, Δ*H_c_* is the cold crystallization enthalpy (J/g) of PLA in the sample, Δ*H^o^_m_* is the heat of fusion for completely crystalline PLA (93.7 J/g) [27], and *w* is the weight fraction of PLA in the samples.

### 3.11. Thermogravimetric Analysis (TGA)

The thermal stability analysis tests of the film were carried out by using Net-Zach DSC-200PC analyzer (Selb, Germany). The samples were sealed in a small ceramic cup and heated from 20 to 600 °C at the speed of 10 °C/min in a nitrogen environment. The weight loss of samples was measured as a function of temperature [51].

### 3.12. Sample Preparation

For the shelf-life test, approximately 20 g of Yunnan “Zhaotong” apple was packaged in individual pouches of different packaging materials. Four groups of samples were prepared in total: PLA/C6 group; PLA/C6/ZnO1% group; PLA/C6/ZnO3% group and PLA group. The samples were stored at 4 ± 1 °C for 14 days. At 0, 2, 4, 6, 8, 10, 12 and 14 days of storage, weight loss, tissue firmness, polyphenoloxidase (PPO), total phenol content, color, microbiological quality, and sensory attributes were analyzed.

### 3.13. Weight Loss

The weight of four apple samples was determined respectively each sampling time, and was compared with the weight on the first day of storage. Weight loss was determined by gravimetry. The weight loss can be expressed as a relative percentage using the following equation:

(4) $$\mathrm{Weight}\;\mathrm{loss}\left(\%\right)=\frac{M_{0}-M_{1}}{M_{0}}\times 100$$

where *M*_0_ was the weight on the first day and *M*_1_ was the weight on each sampling day.

### 3.14. Tissue Firmness Measurement

The firmness of apple samples was evaluated by a penetration test with a texture analyzer (TA-XT, Stable Microsystems, London, UK) equipped with a cylindrical probe of 2 mm diameter. The method was similar to our previous work, and firmness was defined as the maximum force (Newton, N).

### 3.15. Measurement of Polyphenol Oxidase (PPO) Activity

To determine the activity of PPO, fresh-cut apples (5 g) were homogenized with 20 mL ice-cold citric acid buffer (0.2 M, pH 6.8) which contains 20 g/L of polyvinylpyrroline to prevent the oxidation of the samples. Before it was centrifuged at 10,000× *g* for 30 min at 4 °C, the homogenized sample was filtered and kept at 4 °C for 1 h. The collected supernatant was used as a crude enzyme extract. After the extraction, in order to avoid degradation of enzymes, the activity of the PPO was measured instantly. Based on the oxidation of p-phenylendiamine by catechol, PPO activity was assessed. By using the UV-vis Spectrophotometer (T90, Beijing Purkinje General Instrument Co., Ltd., Beijing, China) to measure the absorbance at 398 nm, the increase of absorbance at 398 nm at 25 °C within 2 min was recorded. The results are shown in units of ΔOD_398_/(min g FW) [45].

### 3.16. Total Phenolics

#### 3.16.1. Samples Extraction

A decagram of the samples was homogenized at 4 °C with 40 mL of 80% cold methanol, centrifuged at 10,000× *g* for 20 min and then filtered. The residues were re-extracted twice and supernatant was collected. All of the supernatants were combined for following analyses.

#### 3.16.2. Total Phenolic Measurement

The total phenolic contents of the fresh-cut apples were determined according to the Folin–Ciocalteau method. 3.9 mL distilled water was mixed with 0.1 mL supernatant, and then 0.75 mL sodium carbonate solution and 0.25 mL folin-ciocalteau reagent was added. Before the mixture was incubated for 2 h at room temperature, it was allowed to react in a vortex mixer. A spectrophotometer (UV-1800, Mapada Instruments Co., Ltd., Shanghai, China) was used to measure the absorbance of the mixture. Total phenolic content was expressed as mg gallic acid equivalents (GAE) in 1000 g^−1^ of fresh-cut apples, mg/kg.

### 3.17. Color Measurement

The color of fresh-cut apples was determined by measuring L* (light/dark), a* (red/green), and b* (yellow/blue) using a colorimeter (WSC-S; Shanghai precision instrument Co., Ltd., Shanghai, China). Three color measurements were done at three locations for each sample. In addition, the browning index (BI) was calculated and used as an indicator of brown color intensity. The BI was calculated as:

(5) $$\mathrm{BI}=\frac{100\left(x-0.31\right)}{0.172}$$

where *x* = (a* + 1.75 L*)/(5.645 L* + a* − 3.012 b*).

### 3.18. Microbiological Analysis

According to the plate counting method, the total bacterial count, yeast and fungi counts of the samples can be evaluated. Briefly, a sample of 10 g transferred aseptically into 90 mL of sterile 0.85% (*w*/*v*) NaCl solution and homogenized in a stomacher lab blender. Serial decimal dilutions were prepared in sterile peptone water and pour-plated onto a plate count agar (PCA) and a potato dextrose agar (PDA) plate. The total bacterial count was cultivated on plate count agar (Oxoid, London, UK) at 30 °C for 48 h; yeasts and molds were cultivated on peptone dextrose agar (Oxoid, London, UK) at 30 °C for 48 h. All counts were the average of two different samples and expressed as log cfu/mL.

### 3.19. Sensory Evaluation

The sensory evaluation was carried out by a panel consisting of ten experienced assessors from the Institute of Yunnan Food Safety, Kunming University of Science and Technology (Kunming, China). The test was performed immediately after removal from packaging film. The order of the samples was randomized for each assessor. Odor, color and texture were scored on a nine-point scale where zero equaled “dislike extremely” and nine equaled “like extremely”. For the evaluation of the overall acceptability, a similar scale, where one meant “inedible”, three meant “poor”, five meant “fair” (limit of marketability), seven meant “good” and nine meant “excellent” was used.

### 3.20. Statistical Analysis

All the experiments were conducted in triplicate, and SPSS software (SPSS Inc., version 13.0, Chicago, IL, USA) was utilized to calculate analysis of variance (ANOVA). Significance between mean values was determined by Duncan’s multiple range tests. 0.05 was the significant limit.

## 4. Conclusions

In this work, PLA/ZnO nanocomposites with homogeneously dispersed nanoparticles were prepared by the solvent volatilizing method. The SEM analysis showed that the incompatibility of PLA and the addition of nanoparticles in blends influenced the morphology. DSC and XRD analysis also demonstrated that the nanoblend films were mostly amorphous. The mechanical properties test showed that, with the introduction of ZnO nanoparticles into the PLA, the elongation at break (ε) and elastic modulus (EM) of the nanoblends increased, while tensile strength (TS) decreased. The TGA result showed that the nanoblends had a good thermostability. In addition, compared to the PLA film, the nano-ZnO packaging film has a higher water vapor permeability (WVP) and opacity, and a lower oxygen permeability.

Most importantly, the novel nano-ZnO packaging film was successfully used in the preservation of fresh-cut apple at 4 °C for 14 days. The nano-blend film has a better performance in the maintenance of tissue firmness, total phenolic and the sensory value, and in the reduction of the activity of PPO, as well as in the inhibition of the browning index (BI) and the microbial growth of the fresh-cut apple. The result was that the nano-ZnO packaging film was conducive in maintaining preservation quality of fresh-cut apple.

## Figures and Tables

**Table 1:** Water vapor permeability (WVP) and oxygen permeability of different films. Data are presented as mean ± standard deviation.

Treatment	WVP × 10^−11^(gm/m^2^·s pa)	O_2_ Permeability [(cm^3^/(24 h × m^2^)] × (cm/bar)
PLA	2.033 ± 0.15 ^a^	2.21 ± 0.11 ^b^
PLA/C6	2.323 ± 0.20 ^b^	2.55 ± 0.16 ^c^
PLA/C6/ZnO1%	2.832 ± 0.65 ^c^	1.92 ± 0.13 ^a^
PLA/C6/ZnO3%	2.723 ± 0.38 ^c^	1.84 ± 0.15 ^a^

^a–c^ Values followed by different letters in the same column were significantly different (*p* < 0.05), where ^a^ is the lowest value.

**Table 2:** The mechanical properties of pure PLA and PLA nano-blend films. Data are presented as mean ± standard deviation.

Sample	Elasticity Modulus (EM)	Tensile Strength (TS)	Elongation of Break (%)
PLA/C6/ZnO3%	2528.20 ± 223.54 ^a,b^	14.15 ± 1.55 ^a^	28.40 ± 2.11 ^b^
PLA/C6/ZnO1%	2604.31 ± 297.81 ^b^	18.16 ± 1.69 ^b^	32.22 ± 1.12 ^c^
PLA/C6	2210.64 ± 297.51 ^a^	22.96 ± 2.19 ^c^	27.74 ± 1.57 ^b^
PLA	3027.79 ± 176.41 ^c^	47.78 ± 5.18 ^d^	5.35 ± 0.56 ^a^

^a–d^ Values followed by different letters in the same column were significantly different (*p* < 0.05), where ^a^ is the lowest value.

**Table 3:** Thermal Characteristics of pure PLA and PLA nano-composite films.

Sample	*T_g_* (°C)	*T_c_* (°C)	*T_m_* (°C)	*X_c_* (%)
PLA/C6/ZnO3%	53.0	113.0	171.7	14.3
PLA/C6/ZnO1%	53.1	112.6	165.9	16.2
PLA/C6	52.2	110.7	165.7	7.3
PLA	58.6	109.3	164.2	6.5

**Table 4:** Effect of different packaging on the sensory evaluation of fresh-cut apple during storage at 4 ± 1 °C, and the data are presented as mean ± standard deviation.

Treatments	Odor	Color	Texture	Overall Acceptability
Day 0	9	9	9	9
Day 2	-			
PLA	8.06 ± 0.07 ^a^	7.84 ± 0.05 ^b^	8.04 ± 0.05 ^a^	7.94 ± 0.12 ^a^
PLA/C6	8.14 ± 0.08 ^a^	7.72 ± 0.19 ^b^	8.16 ± 0.15 ^a^	8.01 ± 0.1 ^a^
PLA/C6/ZnO1%	8.16 ± 0.11 ^a^	7.45 ± 0.12 ^a^	8.22 ± 0.04 ^a^	8.13 ± 0.11 ^a^
PLA/C6/ZnO3%	8.15 ± 0.1 ^a^	7.38 ± 0.13 ^a^	8.20 ± 0.14 ^a^	8.15 ± 0.14 ^a^
Day 4	-			
PLA	7.12 ± 0.14 ^a^	7.03 ± 0.14 ^b^	7.20 ± 0.07 ^a^	6.96 ± 0.08 ^a^
PLA/C6	7.24 ± 0.11 ^a,b^	6.95 ± 0.14 ^b^	7.24 ± 0.16 ^a^	7.08 ± 0.15 ^a^
PLA/C6/ZnO1%	7.32 ± 0.06 ^a,b^	6.86 ± 0.12 ^a^	7.28 ± 0.16 ^a^	7.12 ± 0.07 ^a^
PLA/C6/ZnO3%	7.35 ± 0.07 ^b^	6.82 ± 0.05 ^a^	7.3 ± 0.08 ^a^	7.16 ± 0.13 ^a^
Day 6	-			
PLA	5.82 ± 0.04 ^a^	6.43 ± 0.07 ^b^	6.24 ± 0.09 ^a^	5.98 ± 0.06 ^a^
PLA/C6	6.66 ± 0.1 ^b^	6.21 ± 0.06 ^ab^	6.62 ± 0.06 ^b^	6.56 ± 0.13 ^b^
PLA/C6/ZnO1%	6.82 ± 0.05 ^c^	6.18 ± 0.07 ^a^	6.73 ± 0.08 ^b^	6.74 ± 0.09 ^c^
PLA/C6/ZnO3%	6.86 ± 0.12 ^c^	6.15 ± 0.12 ^a^	6.76 ± 0.08 ^b^	6.83 ± 0.04 ^c^
Day 8	-			
PLA	4.78 ± 0.14 ^a^	5.75 ± 0.05 ^b^	5.18 ± 0.09 ^a^	4.86 ± 0.07 ^a^
PLA/C6	5.97 ± 0.12 ^b^	5.63 ± 0.06 ^ab^	6.05 ± 0.1 ^b^	6.01 ± 0.04 ^b^
PLA/C6/ZnO1%	6.24 ± 0.07 ^c^	5.58 ± 0.06 ^a^	6.13 ± 0.04 ^bc^	6.36 ± 0.11 ^c^
PLA/C6/ZnO3%	6.28 ± 0.12 ^c^	5.50 ± 0.11 ^a^	6.24 ± 0.08 ^c^	6.38 ± 0.05 ^c^
Day 10	-			
PLA	4.02 ± 0.13 ^a^	5.26 ± 0.12 ^b^	4.46 ± 0.12 ^a^	4.16 ± 0.16 ^a^
PLA/C6	5.13 ± 0.05 ^b^	4.68 ± 0.47 ^a^	5.69 ± 0.13 ^b^	5.34 ± 0.12 ^b^
PLA/C6/ZnO1%	5.64 ± 0.11 ^c^	5.07 ± 0.11 ^ab^	5.58 ± 0.14 ^b^	5.63 ± 0.09 ^c^
PLA/C6/ZnO3%	5.63 ± 0.18 ^c^	5.08 ± 0.16 ^ab^	5.54 ± 0.08 ^b^	5.61 ± 0.13 ^c^
Day 12				
PLA	3.67 ± 0.11 ^a^	4.83 ± 0.07 ^a^	4.04 ± 0.16 ^a^	3.73 ± 0.18 ^a^
PLA/C6	4.62 ± 0.1 ^b^	4.82 ± 0.04 ^a^	5.04 ± 0.14 ^b^	4.74 ± 0.08 ^b^
PLA/C6/ZnO1%	5.25 ± 0.09 ^c^	4.95 ± 0.09 ^a^	5.13 ± 0.11 ^b^	5.16 ± 0.13 ^c^
PLA/C6/ZnO3%	5.29 ± 0.17 ^c^	4.97 ± 0.1 ^a^	5.21 ± 0.09 ^b^	5.25 ± 0.17 ^c^
Day 14				
PLA	3.31 ± 0.12 ^a^	4.14 ± 0.12 ^a^	3.25 ± 0.13 ^a^	3.44 ± 0.11 ^a^
PLA/C6	4.19 ± 0.15 ^b^	4.27 ± 0.1 ^a^	4.64 ± 0.15 ^b^	4.23 ± 0.12 ^b^
PLA/C6/ZnO1%	4.74 ± 0.09 ^c^	4.33 ± 0.17 ^a^	4.76 ± 0.09 ^b^	5.02 ± 0.09 ^c^
PLA/C6/ZnO3%	4.76 ± 0.11 ^c^	4.36 ± 0.07 ^a^	4.82 ± 0.07 ^b^	5.05 ± 0.09 ^c^

^a–c^ Values followed by different letters in the same column were significantly different (*p* < 0.05), where ^a^ is the lowest value.
